# Left Turns by Older Drivers With Vision Impairment: A Naturalistic Driving Study

**DOI:** 10.1093/geroni/igab026

**Published:** 2021-08-01

**Authors:** Thomas A Swain, Gerald McGwin, Jonathan F Antin, Joanne M Wood, Cynthia Owsley

**Affiliations:** 1 Department of Ophthalmology & Visual Sciences, School of Medicine, University of Alabama at Birmingham Birmingham, Alabama, USA; 2 Department of Epidemiology, School of Public Health, University of Alabama at Birmingham, Birmingham, Alabama, USA; 3 Vulnerable Road User Safety, Virginia Tech Transportation Institute, Blacksburg, Virginia, USA; 4 Centre for Vision and Eye Research, School of Optometry and Vision Science, Queensland University of Technology, Brisbane, Queensland, Australia

**Keywords:** Cognition, Driving, Vision

## Abstract

**Background and Objectives:**

Older drivers are overrepresented in collisions at intersections while making left turns across oncoming traffic. Using naturalistic driving methods, we evaluated the association between vision impairment and their left-turn characteristics.

**Research Design and Methods:**

In this prospective, observational study, vision impairment as defined by visual acuity, contrast sensitivity, visual processing speed, visual field sensitivity, and motion perception was assessed in drivers ≥70 years old. Data acquisition systems were installed in their personal vehicles recording video and vehicle kinematics. Driving during everyday life was recorded for 6 months. Data analysts evaluated a temporal data window surrounding randomly selected left turns at 4-way intersections. Left-turn traversals and turning behavior were evaluated in terms of age-adjusted associations with vision impairment.

**Results:**

The sample consisted of 151 older drivers. The number of turns studied was 473; 265 turns were rated as unsafe traversals, and 201 as problematic turning behavior. Drivers with slowed visual processing speed and visual field impairment were less likely to exhibit unsafe traversals (*p* < .05); those with worse contrast sensitivity, slowed visual processing speed, and visual field impairment were less likely to exhibit problematic turning behavior (*p* < .05).

**Discussion and Implications:**

Using naturalistic driving, our study suggests older drivers with vision impairment exhibit better performance in making left turns than those without deficits, which contradicts older driver studies on left turns using driving simulators and on-road driving evaluations. Our findings suggest more cautious and self-regulatory behavior, which are consistent with older visually impaired drivers’ commonly expressed concerns about their driving difficulties.

**Translational Significance:** In contrast to previous older driver studies using driving simulators and on-road driving evaluations, our study suggests that in everyday life older visually impaired drivers exercise caution in left-turn behavior across oncoming traffic as compared to those who are normally sighted.

Older drivers, aged 65 and older, have a higher risk of collisions at intersections while making left turns across oncoming traffic compared to those aged 16–64 (1) and 25–64 (2). In urban intersections in Minnesota and Illinois, for older drivers aged 75 and older, left-turn collisions represented 60.7% of their collisions, whereas for drivers aged 30–74 only 28.0% of collisions were left turns (3). Similarly, in Orange County, CA, the proportion of collisions involving left turns was higher among adults aged 65+ compared to those 18–64 (4). Possible explanations for older adults’ overrepresentation in left-turn collisions have been explored in the literature, including older adults’ insensitivity to the speed of vehicle approach in the oncoming lane (5,6) and a focus on their own vehicle’s travel path at the expense of ignoring other hazards outside their own vehicle direction (7,8). Older drivers, as compared to young drivers, tend to underestimate a vehicle’s velocity in an oncoming lane at higher speeds (6). It has been argued that their inaccurate judgment of vehicle speed in the oncoming lane may thwart their final judgment on executing a turn (9). These decisions rely on visual and visuo-cognitive based decisions indicating functional ability could be a contributing factor to why older adults have increased left-turn collision risk.

In questionnaire studies, older adults self-report driving difficulty with, and avoidance of, certain roadway scenarios including left turns (10–12). Furthermore, their reported difficulties in certain driving environments have been associated with vision impairment and diagnosed eye disease. Drivers with glaucoma report restricting their driving at night, on freeways, and in unfamiliar areas (13), and attribute their driving limitations to problems with their vision (14). In a cross-sectional study, worse contrast sensitivity in older drivers was associated with a reported reduction in driving exposure (15). This study further showed that worse contrast sensitivity and light sensitivity in the central and peripheral visual fields were associated with night driving cessation in the subsequent 2 years (16). A study on older drivers with cataract showed that drivers with deficits in visual acuity and contrast sensitivity were more likely to express difficulty with many driving maneuvers; for left turns, drivers with worse than 20/50 acuity had a 9.5-fold reported increase in left-turn difficulty and for those with log contrast sensitivity of 1.25 or worse, a 13-fold increase in left-turn difficulty (17). In addition, recent work on left-turn collision risk factors using naturalistic driving data in older drivers indicates that health and cognitive factors had stronger associations to crashes during left turns than did intersection infrastructure (18).

Despite the link between vision impairment and older adults’ self-reported difficulty in executing left turns and prior work indicating associations between visual function and driving performance in simulator and on-road evaluations (19–26), no study using naturalistic driving methods has specifically focused on the relationship between vision impairment in older drivers and actual driving behaviors during left turns. A previous study using naturalistic driving methods focused on older drivers’ intersection behaviors including turning; however, only 10 drivers were studied for 2 weeks, all of whom had good vision (27). By naturalistic methods, we mean measurement methods in which participant drivers have a data acquisition system (DAS) with multichannel synchronized video and sensors installed in their own vehicles (28). Older drivers in our study then drove as they normally would for 6 months. Most, but not all, drivers had aging-related eye conditions that could impair vision. Prior to system installation, we measured several aspects of visual function. Our hypothesis was that there is a significant association between vision impairment and unsafe and problematic left-turn behaviors among older drivers.

## Method

### Study Design and Sample

Data were collected in the Alabama VIP Older Driver study, a 6-month prospective cohort study on adults aged ≥70 years of age utilizing naturalistic driving methods. The details of the study design have been published elsewhere (28,29). Briefly, participants were recruited due to a recent visit at the Callahan Eye Hospital’s clinics at the University of Alabama at Birmingham (UAB) and the electronic health record. Recruitment focused on those with eye conditions with the potential for vision impairment to ensure a sample with varied visual function (unimpaired to impaired). Prior to enrollment, participants were contacted via a letter and a telephone screening call to schedule an in-person visit. Inclusion criteria were participants had to be 70 years of age or older, own a vehicle compatible with the DAS (vehicle model years older than 1996 were ineligible), be legally licensed to drive in Alabama, report driving at least 4 days per week, and be willing to have the DAS installed in their vehicle. Those meeting all inclusion criteria but who had planned periods of 2 consecutive weeks or more of no driving (eg, vacation, planned hospitalization) were excluded from participation. Written informed consent was obtained prior to enrollment. This study was approved by the institutional review boards at UAB and Virginia Tech and followed the tenets of the Declaration of Helsinki.

### Baseline Assessments

After enrollment, participants completed the baseline visit consisting of DAS installation in their vehicles while they underwent the baseline assessment. Demographic information (eg, date of birth, sex, race, education level) was obtained by interview. Participants completed a battery of visual and visuo-cognitive tests, all under photopic and binocular conditions unless otherwise noted. Habitual distance visual acuity was measured under the standard protocol of the Electronic Visual Acuity expressed as the logarithm of the minimum angle of resolution (logMAR) (30). Those with visual acuity greater than 0.3 logMAR (worse than 20/40) were considered impaired, corresponding to the definition of impairment used by many governmental jurisdictions for driver licensing requirements. Contrast sensitivity was assessed using the Pelli–Robson chart (31), scored by the letter-by-letter method (32), and expressed as log sensitivity. Impaired contrast sensitivity was defined as <1.5 log sensitivity as per previous work (33).

The Humphrey Field Analyzer (HFA), Model II-I, was used to assess visual field sensitivity, measured in decibels (dB). A custom test with 20 white stimulus-size III targets using a full threshold procedure was implemented as described previously (34,35). Visual field sensitivity was assessed monocularly, so the HFA’s fixation tracking could be utilized. Monocular sensitivities were combined to create binocular field sensitivities. The sensitivity at each test location was represented by the most sensitive measurement (ie, highest value) of the 2 eyes (36). The area of the binocular field extended 15° superiorly, 30° inferiorly, and 60° horizontally, to correspond to viewing the roadway environment through a windshield as well as the upper dashboard (37). For the 2 sensitivities measured temporally at 60° along the horizontal meridian, the sensitivity of each eye defined the binocular visual field sensitivity. In addition to expressing the overall visual field sensitivity as the average of all points, the average sensitivity was also calculated for various driving visual field regions including superior (all test locations above the horizontal meridian), inferior (all test locations below the horizontal meridian), left (all test locations to the left of the vertical meridian), right (all test locations to the right of the vertical meridian), and peripheral (test locations at or temporal to ±45°). Impairment for each visual field sensitivity region was defined by the first dB quartile (34).

Motion perception threshold was evaluated using the drifting Gabor test (38). In this test, the participant was presented with a 3 cycle/degree vertical sinusoidal grating filtered with a Gaussian envelope. Participants were asked to identify the direction of the grating (right vs left) with the drift rate (hertz, Hz) varying during a 2-down/1-up staircase with 8 reversals. The threshold was calculated as the average of the last 6 reversals and expressed in Hz. Impaired motion perception was defined by the median value, with those below the median considered to be impaired (39).

Visual processing speed was assessed using the useful field of view subtest 2 (40). This test measures the time in milliseconds (ms) for a participant to discriminate 2 targets in central vision while simultaneously localizing a peripheral target (10° eccentricity) at any of 8 radial directions. Processing speeds were defined as moderately (150–350 ms) and severely (>350 ms) impaired (41). The Trail Making Test part B (Trails B) was also used to assess visual processing speed in combination with executive function and working memory (42). In this test, the time in minutes is measured for the participant to draw a line from numbers and letters alternately, following the numerical and alphabetical order. Trails B times ≥ 2.47 minutes were considered impaired (43). The Visual Closure Subtest of the motor-free visual perception version 3 test was used to evaluate spatial ability (44). This test presents cards of objects drawn both incompletely and completely, and participants are asked to match the 2 versions. Impairment was defined as scores < 8 per previous work (45).

Evaluations were also completed to assess general cognitive status, depressive symptoms, and general health. The Mini-Mental State Screening Examination (MMSE) assessed general cognitive status (46), and the Center for Epidemiological Studies—Depression scale (CES-D) assessed depressive symptoms (47). Those with MMSE scores less than 26 were considered impaired. Generally, persons with MMSE scores less than 24 are considered cognitively impaired (46); however, due to the very low number of persons in the cohort meeting this definition of impairment, we utilized 26 and below, representing the first quartile as impaired. CES-D scores greater than 16 are considered depressed (47). General health was assessed via interview where participants were asked about medical problems in 17 areas (eg, heart disease, diabetes, cancer) (48).

After the baseline visit and installation of the DAS was complete, participants then drove their vehicle as they normally would in everyday life for a period of 6 months. The DAS was developed by the Virginia Tech Transportation Institute (VTTI) and was the same system used in the Strategic Highway Research Program (SHRP2) naturalistic driving study (49). The DAS recorded various data streams including: 5-channel video (views of 83° forward, 55° left side; 99° rear; 99° front seat passenger/cabin snapshot; 55° of steering wheel and driver controls), accelerometers, global positioning system (GPS), and vehicle network information (eg, speed, engine revolutions per minute, brake actuation). The DAS turned on/off with the vehicle and recorded data to a hard drive. During the follow-up driving period, the DAS was checked remotely by VTTI through cell phone networking to identify potential operational problems. If problems arose, study staff contacted the participant, so their vehicle and DAS could undergo maintenance, repair, or replacement. After the 6-month prospective driving period, the DAS was deinstalled from the participant vehicle and data were transmitted from UAB to VTTI for processing and reduction.

### Turn Identification, Selection, and Data Reduction

To identify left turns during the 6-month follow-up period, data reduction analysts at VTTI used accelerometer data in conjunction with GPS coordinates to determine all left turns occurring during the follow-up period. Turn frequency and location were synchronized with maps (Esri, ArcGIS). Our focus was on left turns at 4-way intersections with any of the following traffic control devices: traffic light, stop sign, yield sign. To select turns occurring at locations meeting the criteria, turns were randomized by participant and the location assessed for selection criteria via map and street view on Google Maps. The first 3 turns for each participant meeting criteria were selected for data reduction. A fourth turn meeting criteria was selected from a random subselection of participants to obtain 500 total turns for data reduction. Each turn was assessed by 2 trained analysts, one who completed the reduction and another who checked the initial assessment. If a disagreement was noted by the second analyst, the 2 met and reconciled the issue. Any disagreements between analysts were reviewed by a trained supervisor.

Analysts viewed a temporal segment of video surrounding the left-turn events and coded the following factors: intersection type (eg, 4-way perpendicular, 4-way skewed, T-intersection, Y-intersection), type of traffic control (eg, stop sign, yield sign, traffic signal), lighting (eg, dawn, daylight, dusk, darkness [lighted], darkness [not lighted]), weather (eg, clear/partly cloudy, overcast, mist/light rain, raining), and front seat passenger presence versus absences.

Analysts coded 2 left-turn outcomes of interest: (i) safe traversal versus unsafe traversal through the intersection, (ii) normal versus problematic turn behavior. SHRP2 methods developed to examine aspects of safety critical event reductions served as the basis of the items noted and included in these outcomes (50). Unsafe traversal was defined as any left turn in which the analyst judged that the driver passed through the intersection in a manner that posed a threat to roadway safety. Any of the following nonmutually exclusive characteristics of the video coded by analysts defined unsafe traversal: late or no turn signal, followed too closely, turned from wrong lane, misjudged traffic flow, jerky movement through intersection, poor lane keeping, too fast, violated traffic control device, and wrong destination lane. Late or no turn signal was defined as activating the turn signal after entering the intersection or not signaling. Drivers traveling through the intersection with less than 2 seconds of headway were noted as following too closely. Turns occurring from a lane not intended for making the turn were categorized as turns from the wrong lane. Drivers who misperceived the direction of travel or speed of oncoming traffic were considered to have misjudged traffic flow. Jerky movement was designated if the participant’s vehicle demonstrated one or more sudden braking or steering events during the turn, unwarranted by other traffic or scenario-specific circumstances. Poor lane keeping was designated if the subject vehicle strayed unnecessarily from the normal or designated path during the turn. Too fast was designated if the subject vehicle was judged to take the turn at a higher rate of speed than safely supported by intersection configuration, traffic density, or other factors (eg, weather). Violation of a traffic control device was noted when the driver did not observe the traffic control device (eg, no stop, rolling stop). Wrong destination lane was defined for turns where the driver did not travel to the correct lane while completing their turn (ie, did not travel in lane closest from origin).

Problematic left-turn behavior was defined as driving behaviors that violated sound driving norms for left turns. These behaviors including any of the following nonmutually exclusive characteristics: lane drifting, right-of-way error, sudden/improper braking, too slow, turned from wrong lane, turned tight, turned wide, lane change with no warning, violation of traffic control device, and driving on the wrong side of road. Lane drifting was defined as the inability to maintain appropriate and safe lane position and unintentionally drifts toward or over one or more lanes. Turns where the driver made the incorrect decision with regard to who had the right-of-way were considered a right-of-way error. Sudden/improper braking was noted when the driver braked suddenly or in an unsafe manner in the roadway but did not come to a full stop. Too slow was defined as the driver traveling at a speed much lower than the posted speed limit when higher speeds are appropriate (eg, traveling 10 mph under posted speed limit). Instances in which the driver unnecessarily encroached onto the right adjacent lane, shoulder, or curb were considered turning wide. Similarly, instances in which the driver unnecessarily encroached onto the left adjacent lane, shoulder, or curb were considered turning tight. Lane change with no warning occurred when the driver failed to or delayed use of the turn signal. Turns made where the driver was traveling on the wrong side of the road were considered as occurring on the wrong side of the road. Turning from the wrong lane and violation of traffic control device were defined for turn behavior as under unsafe traversal. Analysts were masked to all participants’ visual and other characteristics collected at UAB.

### Statistical Analysis

VTTI transmitted the left-turn data to UAB for statistical analysis. Participant IDs were used to link left-turn variables to participant’s demographic, visual function, and other information collected at baseline. Generalized estimating equations (GEEs) were used to calculate odds ratios (ORs) and 95% confidence intervals (95% CIs) for the 2 outcomes of interest with each categorical visual function measure considered independently. GEE models were used to account for the fact that participants could have more than 1 turn. Confounding was assessed for gender, race, MMSE score, CES-D score, years of education, number of medical conditions, lens status, glaucoma, diabetic retinopathy/macular edema, and age-related macular degeneration; however, none was detected. ORs are age-adjusted. The level of significance was set at <.05 (2-sided), and all analyses were completed in SAS Version 9.4 (SAS Institute, Cary, NC).

## Results

There were 321 drivers meeting eligibility criteria after completing a telephone screening. Of these, 280 completed an in-person screening visit, of whom 162 met inclusion criteria and passed vehicle screening and were invited to enroll. The total number of drivers who enrolled in the Alabama VIP study was 159. Of these, 5 drivers dropped out shortly after the DAS was installed because the DAS caused vehicle inconvenience (eg, radio static). Of the 154 who continued in the study, 8 drivers did not complete the full 6-month follow-up and terminated the study due to various reasons (eg, vehicle needed repair, a serious medical issue prevented driving). However, they were included in the analysis because on average they drove for >3 months of the 6-month follow-up period. Of the 154 drivers, left turns were not identifiable for 1 participant due to GPS malfunctions. In all, 54 476 left turns were identified among 153 drivers during the follow-up. Of the 500 left turns randomly selected for data reduction for 153 participants, it was later determined at further visual inspection that 27 turns did not occur at a 4-way intersection and/or there was no traffic control device, so these turns did not meet the study’s left-turn criteria. This eliminated 2 additional drivers from the sample. The final analysis sample consisted of 473 turns completed among 151 participants. The majority of turns occurred during daylight (92.6%) and under clear/partly cloudy conditions (80.1%). Front seat passengers were present during 27.3% of turns.

Of the 151 drivers in the sample, participants drove on average 3 897 ± 4 402 miles during the follow-up period. There were 35 participants (23.2%) with 4 turns, 102 (67.6%) with 3 turns, 13 (8.6%) with 2 turns, and 1 (0.7%) with 1 turn. The majority of the study participants were in their 70s (55.6%) or their 80s (42.4%), with a mean age of 79.2 years (standard deviation 5.1; Table 1). There were more male (55%) than female drivers. Most of the sample completed high school or greater (15 ± 2.7 years), were not cognitively impaired (MMSE score: 27.8 ± 1.8), and were not depressed (CES-D score: 3.8 ± 4.3). The majority of participants (59%) had 4 or more medical conditions and 91.2% had an eye-related condition. Of the sample, 22.5% had no visual impairment, 23.2% had 1 visual impairment, with the remainder having 2 or more impairments, indicating a range of visual function within the sample.

**Table 1.** Demographic and Health Characteristics of Participants in the Sample (*N* = 151)

Characteristic	*n* (%)	Mean (*SD*)
Age group, years		
70–79	84 (55.6)	
80–89	64 (42.4)	
90–99	3 (2.0)	
Sex		
Women	68 (45.0)	
Men	83 (55.0)	
Race		
Black	28 (18.5)	
White	123 (81.5)	
Education category		
Less than high school graduate	5 (3.3)	
High school graduate	74 (49.0)	
College graduate	61 (40.4)	
Professional or graduate school	11 (7.3)	
MMSE		
≥26 (not impaired)	113 (74.8)	
<26 (impaired)	38 (25.2)	
Medical conditions, number		
0–1	20 (13.3)	
2–3	42 (27.8)	
4–5	59 (39.1)	
≥6	30 (19.9)	
CES-D		
≤16 (not depressed)	148 (98.0)	
>16 (depressed)	3 (2.0)	
Eye diagnoses (one or both eyes)*		
Age-related macular degeneration	28 (18.9)	
Cataract	62 (41.9)	
Diabetic retinopathy or macular edema	10 (6.8)	
Primary open angle glaucoma	43 (29.1)	
Pseudophakia	91 (61.5)	
Other†	66 (44.6)	
Age, years		79.2 (5.1)
Education, years		15.0 (2.7)
MMSE, total score		27.8 (1.8)
CES-D, total score		3.8 (4.3)

*Notes*: CES-D = Center for Epidemiological Studies—Depression scale; MMSE = Mini-Mental State Examination; *SD* = standard deviation.

*Participants could have more than 1 diagnosis. Medical records could not be located for 3 participants.

^†^Examples of other conditions include dry eye disease, hypertensive retinopathy, Fuchs’ dystrophy, lattice degeneration, macular cyst hole, macular pucker, ocular prosthesis, and retinal tear.

Visual acuity was worse than 20/40 in only 2 participants (1.3%) and contrast sensitivity was impaired in 15 (9.9%; Table 2). Visual processing speed under divided attention was moderately (150–350 ms) or severely impaired (>350 ms) in 49.2% of the sample. Trails B times were impaired in 55 (36.4%) of participants. Overall visual field sensitivity scores were on average 23.9 ± 2.9 dB, with the lowest quartile defined by 22.4 dB. The lowest quartile ranged from 19.3 dB to 22.4 dB for all visual field subregions. Motion perception impairment was defined by worse than the median, 0.14 Hz.

**Table 2.** Visual Function of Participants in the Sample (*N* = 151)

Visual Function	*n* (%)
Visual acuity, logMAR	
≤0.3 (better)	149 (98.7)
>0.3 (worse)	2 (1.3)
Contrast sensitivity, log sensitivity	
≥1.5 (better)	136 (90.1)
<1.5 (worse)	15 (9.9)
UFOV subtest 2,* ms	
<150 (better)	62 (50.8)
150–350	45 (36.9)
>350 (worse)	15 (12.3)
Trails B, minute	
<2.47 (better)	96 (63.6)
≥2.47 (worse)	55 (36.4)
MVPT3, score	
≥8 (better)	140 (92.7)
<8 (worse)	11 (7.3)
Visual field sensitivity, dB, 3 upper quartiles vs lowest quartile	
Overall	
>22.4 (better)	115 (76.2)
≤22.4 (worse)	36 (23.8)
Peripheral (≥ 45°)	
>19.3 (better)	113 (74.8)
≤19.3 (worse)	38 (25.2)
Superior	
>22.2 (better)	110 (72.9)
≤22.2 (worse)	41 (27.2)
Inferior	
>22.1 (better)	112 (74.2)
≤22.1 (worse)	39 (25.8)
Left	
>21.7 (better)	111 (73.5)
≤21.7 (worse)	40 (26.5)
Right	
>21.9 (better)	110 (72.9)
≤21.9 (worse)	41 (27.2)
Gabor drifting grating threshold, Hz, median split	
<0.14 (better)	77 (51.0)
≥0.14 (worse)	74 (49.0)

*Notes*: logMAR = logarithm of the minimum angle of resolution; MVPT3 = motor-free visual perception test; UFOV, useful field of view.

*122 participants completed this testing.

Unsafe left-turn traversals occurred during 265 of 473 turns (56%), and problematic left-turn behavior during 201 of 473 turns (42%) (Table 3). For unsafe traversals, items noted, but which were not mutually exclusive, included poor lane keeping and no turn signal, which occurred in 29% and 18.8% of turns, respectively. Turning tight (27.1%) and turning wide (12.7%), both alone and noted with other issues, are examples of commonly noted problematic behavior at turns.

**Table 3.** Age-Adjusted Visual Function Associations With Driving Events Assessed at Randomly Chosen Left Turns During the Course of the 6-Month Prospective Naturalistic Driving Study (*N* left turns = 473)

Visual Function	Unsafe Traversal vs Safe Traversal (*N* events = 265)		Problematic Behavior vs Normal Behavior (*N* events = 201)	
	OR (95% CI)*	*p* Value	OR (95% CI)*	*p* Value
Contrast sensitivity, log sensitivity				
≥1.5 (better)	REF†		REF	
<1.5 (worse)	0.48 (0.21–1.11)	.088	0.36 (0.18–0.72)	.004
UFOV subtest 2,‡ ms				
<150 (better)	REF		REF	
150–350	1.54 (0.71–3.37)	.276	1.49 (0.70–3.15)	.296
>350 (worse)	0.91 (0.56–1.49)	.719	0.84 (0.50–1.43)	.526
Trails B, minute				
<2.47 (better)	REF		REF	
≥2.47 (worse)	0.54 (0.34–0.85)	.007	0.51 (0.32–0.81)	.004
MVPT3, score				
≥8 (better)	REF		REF	
<8 (worse)	0.79 (0.39–1.61)	.515	0.57 (0.29–1.13)	.109
Overall visual field sensitivity, dB				
>22.4 (better)	REF		REF	
≤22.4 (worse)	0.58 (0.36–0.94)	.026	0.63 (0.38–1.05)	.078
Peripheral visual field sensitivity, dB				
>19.3 (better)	REF		REF	
≤19.3 (worse)	0.48 (0.29–0.79)	.004	0.47 (0.28–0.80)	.005
Upper visual field sensitivity, dB				
>22.2 (better)	REF		REF	
≤22.2 (worse)	0.65 (0.41–1.01)	.055	0.49 (0.30–0.80)	.004
Lower visual field sensitivity, dB				
>22.1 (better)	REF		REF	
≤22.1 (worse)	0.54 (0.34–0.86)	.010	0.55 (0.33–0.90)	.017
Left visual field sensitivity, dB				
>21.7 (better)	REF		REF	
≤21.7 (worse)	0.52 (0.32–0.84)	.007	0.49 (0.29–0.81)	.005
Right visual field sensitivity, dB				
>21.9 (better)	REF		REF	
≤21.9 (worse)	0.67 (0.43–1.05)	.080	0.68 (0.41–1.11)	.124
Gabor drifting grating threshold, Hz				
≤0.14 (better)	REF		REF	
>0.14 (worse)	0.73 (0.49–1.09)	.120	0.67 (0.43–1.05)	.079

*Notes*: MVPT3 = motor-free visual perception test; UFOV, useful field of view.

*Odds ratio (OR) and 95% confidence interval (95% CI).

^†^Reference group.

^‡^The association between UFOV and the left-turn outcomes had reduced sample size because 122 drivers were assessed for UFOV. They had 384 total turns. For unsafe traversal, they had 214 turns and for turn behavior, 165 turns.

Because only 2 of 151 drivers in our sample had visual acuity worse than 20/40, we did not evaluate the association between impaired acuity and left-turn behaviors. With respect to unsafe left-turn traversals, those with slower Trails B times (meaning slower visual processing speed also relying on executive function and working memory) were 46% less likely to have an unsafe traversal compared to those with faster Trails B times (OR: 0.54, 95% CI: 0.34–0.85). Those with impaired overall, peripheral, lower, and left visual field sensitivities were all at reduced odds of unsafe traversal (OR: 0.58, 95% CI: 0.36–0.94; OR: 0.48, 95% CI: 0.29–0.79; OR: 0.54, 95% CI: 0.34–0.86; OR: 0.52, 95% CI: 0.32–0.84, respectively). Other associations between types of vision impairment and unsafe traversals in left turns were not significant.

With respect to problematic left-turn behavior, drivers with worse contrast sensitivity had 64% reduced odds of problematic turn behavior (OR: 0.36, 95% CI: 0.18–0.72) and those with slower Trails B times had 49% reduced odds of problematic turn behavior (OR: 0.51, 95% CI: 0.32–0.81). Drivers with impairment in the specific regions of the visual field including peripheral, upper, lower, and left areas were also at reduced odds of problematic turn behavior (OR: 0.47, 95% 0.28–0.80; OR: 0.49, 95% CI: 0.30–0.80; OR: 0.55, 95% CI: 0.33–0.90; OR: 0.49, 95% CI: 0.29–0.81, respectively). Other associations between types of vision impairment and problematic left-turn behavior were not significant.

## Discussion

In this naturalistic driving study, older drivers with specific visual and visual–cognitive impairments were more likely to exhibit appropriate left-turn behaviors and traversals through 4-way intersections with traffic control devices as compared to those with unimpaired vision. Specifically, those with visual field impairment, contrast sensitivity, and slowed visual processing speed exhibited fewer errors at left turns than did those without these deficits. This could be viewed as contradictory to findings from existing older driver performance studies in that prior studies suggest that older drivers’ visual deficits are associated with driving problems, not with safer driving performance advantages (19–26). Studies using driving simulators have reported associations between vision impairment and driving performance problems. For example, drivers with central vision impairment due to contrast sensitivity and/or acuity deficits exhibited several performance challenges, including more lane boundary crossings, lateral position errors, scanning deficits in intersections, hazard perception errors, and greater steering corrections compared to normally sighted drivers (19–23). In addition, older driver studies assessing on-road driving performance along a standard route by a professional driving rehabilitation specialist indicated that these drivers made driving errors (unsafe driving maneuvers in intersections and when yielding, problems in lane keeping and making turns) that were associated with vision impairment (24–26). However, through our use of naturalistic driving methods, where older adults perform as they routinely drive in everyday life in their very own vehicle (not in a simulator or test vehicle), the left-turn behaviors of visually impaired drivers were actually better than those drivers who are normally sighted. While we have only studied one type of driving maneuver—left turns—our finding highlights the importance of clarifying the validity of driver safety and performance by visually impaired persons in on-road evaluations and in simulators by comparing it to how driving behavior naturally occurs through naturalistic driving techniques. Given the differing results from this and prior work, our finding can also be viewed as potentially challenging the conventional wisdom that on-road driving evaluations as assessed by a rehabilitation specialist or as implemented in simulators validly represent older drivers’ everyday driving performance.

Our results could be consistent with the widespread literature on self-reported driving difficulties by older drivers. As previously discussed, many studies have shown that older drivers with vision impairment and eye conditions were more likely to report that they are aware of their driving challenges (10,11,13–17). Instead of causing left-turn difficulties, this may create a greater self-awareness in these visually impaired older drivers that prompts more careful and cautious driving behaviors on the road. All our participants were recruited through eye clinics and were most likely aware of eye conditions they had and the visual compromises they could face on the road. Thus, they may have acted on this knowledge in implementing specific driving strategies behind the wheel.

Several studies have evaluated the efficacy of group-administered or individually tailored educational programs for older drivers to assess whether they promote knowledge about safe driving strategies for older drivers (51–55), including those with vision impairment (52,55). These programs improved knowledge and self-regulatory driving strategies and increased avoidance of challenging driving situations. For example, a randomized clinical trial was focused on visually impaired older drivers with visual acuity impairment or slowed visual processing speed; the study implemented an individually tailored educational program addressing how each participant’s visual problems could affect driving performance and safety (56). The program successfully improved self-reported driving strategies for older drivers with vision impairment (52). The findings in the current naturalistic driving study are consistent with the notion that older drivers’ self-awareness about visual function and eye health could practically engender their implementing safe driving practices on the road.

An important question that our study did not investigate is the relationship between our results here and the widely reported findings that various types of vision impairments are risk factors for crash involvement by older drivers (57). Although we have provided data that document older drivers with vision impairment display safer left-turn behaviors, the literature points to these very same risk factors (eg, slowed processing speed, contrast sensitivity deficits, visual field impairment) as elevating collision risk (29,34,58–60). A working hypothesis is that those older drivers who are visually impaired yet who are unaware of their visual limitations may not exercise sufficient caution in on-road behaviors, which may underlie the vision-impairment/crash risk association. This is a question well-suited for naturalistic driving research.

Strengths of our study include the use of naturalistic driving techniques with an instrumented vehicle with video which is an unobtrusive method to study actual left-turn behavior in older drivers with vision impairment. VTTI analysts were masked to all vision and other health characteristics of drivers. All associations between vision impairment and left-turn behaviors (except one) had OR point estimates in the direction of a protective association. Our finding that visually impaired drivers were less likely to engage in problems in left turns is consistent with the large self-report literature that these drivers are aware of their driving limitations. Limitations should also be addressed. Other visual functions known to elevate motor vehicle collision risk in older drivers such as reductions in impaired motion perception were not associated with left-turn behaviors although they elevate crash risk in older drivers (39). The lack of this association may stem from a relatively small sample of 151 drivers that reduced statistical power. We only had 2 participants with visual acuity worse than 20/40, so we were unable to evaluate the association between acuity and left-turn behaviors. Although our study design focused on common aging-related eye conditions, we did not have sufficient sample size to probe left-turn behavior as a function of eye diagnosis. In future naturalistic studies this information will be useful in stratifying visually impaired older drivers into those who have self-awareness about visual limitations on the road versus those who do not. Such research could shed light on how actual driving behaviors, attitudes and beliefs on the part of the driver, and crash involvement may be related.

In summary, as assessed by naturalistic driving techniques, visually impaired older drivers in this study were less likely to engage in problematic performance at left turns at 4-way intersections with traffic control devices. Although this finding may seem paradoxical because driving depends critically on vision, it is consistent with visually impaired drivers’ commonly expressed concerns about their driving difficulties, which may have prompted their more cautious and self-regulatory behavior. Future naturalistic driving research with visually impaired older drivers will be important in further clarifying this finding, and will also reveal other types of driving characteristics displayed by visually impaired older drivers. This work will also clarify to what extent these behaviors are related to elevations or reductions in crash risk. These studies could be informative in designing interventions to enhance road safety in this population.

## References

[1] Chandraratna S, Stamatiadis S. Problem driving maneuvers of elderly drivers. Transp Res Record. 2003;1843:89–95. doi:10.3141/1843-11

[2] Matthias JS, De Nicholas ME, Thomas GB. A study of the relationship between left turn accidents and driver age in Arizona. Phoenix, AZ: Arizona Department of Transportation; 1996.

[3] Federal Highway Administration. Accident Analysis of Older Drivers at Intersections. Washington, DC: US Department of Transportation; 1995.

[4] Lotfipour S, Sayegh R, Chakravarthy B, et al. Fatality and injury severity of older adult motor vehicle collisions in Orange County, California, 1998-2007. West J Emerg Med. 2013;14(1):63–68. doi:10.5811/westjem.2011.8.6610 23451291 PMC3583287

[5] Staplin L. Simulator and field measures of driver age differences in left-turn gap judgments. Transp Res Rec. 1995:1485:49–55.

[6] Scialfa CT, Guzy LT, Leibowitz HW, Garvey PM, Tyrrell RA. Age differences in estimating vehicle velocity. Psychol Aging. 1991;6(1):60–66. doi:10.1037//0882-7974.6.1.60 2029369

[7] Romoser MR, Pollatsek A, Fisher DL, Williams CC. Comparing the glance patterns of older versus younger experienced drivers: scanning for hazards while approaching and entering the intersection. Transp Res Part F Traffic Psychol Behav. 2013;16:104–116. doi:10.1016/j.trf.2012.08.004 23148130 PMC3494462

[8] Hasher L, Zacks RT, May CP. Inhibitory control, circadian arousal, and age. In: Gopher D, Koriat A, eds. Attention and Performance: XVII. Cognitive Regulation of Performance: Interaction of Theory and Application. Cambridge, MA: MIT Press; 1999:653–675. doi:10.7551/mitpress/1480.003.0032

[9] Zhou H, Lownes NE, Ivan JN, Garder PE, Ravishanker N. Left-turn gap acceptance behavior of elderly drivers at unsignalized intersections. J Transpor Safety Security. 2015;7:324–344. doi:10.1080/19439962.2014.976689

[10] Owsley C, Stalvey BT, Phillips JM. The efficacy of an educational intervention in promoting self-regulation among high-risk older drivers. Accid Anal Prev. 2003;35(3):393–400. doi:10.1016/s0001-4575(02)00016-7 12643956

[11] Stalvey BT, Owsley C, Stalvey BT, Owsley C. Self-perceptions and current practices of high-risk older drivers: implications for driver safety interventions. J Health Psychol. 2000;5(4):441–456. doi:10.1177/135910530000500404 22049188

[12] Charlton JL, Oxley J, Fildes B, et al. Characteristics of older drivers who adopt self-regulatory driving behaviours. Transp Res Part F Traffic Psychol Behav. 2006;9:363–373. doi:10.1016/j.trf.2006.06.006

[13] Adler G, Bauer MJ, Rottunda S, Kuskowski M. Driving habits and patterns in older men with glaucoma. Soc Work Health Care. 2005;40(3):75–87. doi:10.1300/J010v40n03_05 15837669

[14] Ramulu PY, West SK, Munoz B, Jampel HD, Friedman DS. Driving cessation and driving limitation in glaucoma: the Salisbury Eye Evaluation Project. Ophthalmology. 2009;116(10):1846–1853. doi:10.1016/j.ophtha.2009.03.033 19592110 PMC2757455

[15] Keay L, Munoz B, Turano KA, et al. Visual and cognitive deficits predict stopping or restricting driving: the Salisbury Eye Evaluation Driving Study (SEEDS). Invest Ophthalmol Vis Sci. 2009;50(1):107–113. doi:10.1167/iovs.08-2367 18719088 PMC2633220

[16] Freeman EE, Muñoz B, Turano KA, West SK. Measures of visual function and their association with driving modification in older adults. Invest Ophthalmol Vis Sci. 2006;47(2):514–520. doi:10.1167/iovs.05-0934 16431944

[17] McGwin G Jr, Chapman V, Owsley C. Visual risk factors for driving difficulty among older drivers. Accid Anal Prev. 2000;32(6):735–744. doi:10.1016/s0001-4575(99)00123-2 10994600

[18] Zafian T, Ryan A, Agrawal R, Samuel S, Knodler M. Using SHRP2 NDS data to examine infrastructure and other factors contributing to older driver crashes during left turns at signalized intersections. Accid Anal Prev. 2021;156:106141. doi:10.1016/j.aap.2021.106141 33873135

[19] Szlyk JP, Pizzimenti CE, Fishman GA, et al. A comparison of driving in older subjects with and without age-related macular degeneration. Arch Ophthalmol. 1995;113(8):1033–1040. doi:10.1001/archopht.1995.01100080085033 7639654

[20] Uc EY, Rizzo M, Anderson SW, Dastrup E, Sparks JD, Dawson JD. Driving under low-contrast visibility conditions in Parkinson disease. Neurology. 2009;73(14):1103–1110. doi:10.1212/WNL.0b013e3181bacf6e 19805726 PMC2764395

[21] Bowers AR, Bronstad PM, Spano LP, Goldstein RB, Peli E. The effects of age and central field loss on head scanning and detection at intersections. Transl Vis Sci Technol. 2019;8(5):14. doi:10.1167/tvst.8.5.14 PMC6753881 31588377

[22] Bronstad PM, Bowers AR, Albu A, Goldstein R, Peli E. Driving with central field loss I: effect of central scotomas on responses to hazards. JAMA Ophthalmol. 2013;131(3):303–309. doi:10.1001/jamaophthalmol.2013.1443 23329309 PMC3605225

[23] Bronstad PM, Albu A, Goldstein R, Peli E, Bowers AR. Driving with central field loss III: vehicle control. Clin Exp Optom. 2016;99(5):435–440. doi:10.1111/cxo.12432 27452786 PMC5542065

[24] Uc EY, Rizzo M, O’Shea AMJ, Anderson SW, Dawson JD. Longitudinal decline of driving safety in Parkinson disease. Neurology. 2017;89(19):1951–1958. doi:10.1212/WNL.0000000000004629 29021353 PMC5679414

[25] Wood JM, Anstey KJ, Kerr GK, Lacherez PF, Lord S. A multidomain approach for predicting older driver safety under in-traffic road conditions. J Am Geriatr Soc. 2008;56(6):986–993. doi:10.1111/j.1532-5415.2008.01709.x 18422946

[26] Wood JM, Mallon K. Comparison of driving performance of young and old drivers (with and without visual impairment) measured during in-traffic conditions. Optom Vis Sci. 2001;78(5):343–349. doi:10.1097/00006324-200105000-00018 11384012

[27] Charlton JL, Catchlove M, Scully M, Koppel S, Newstead S. Older driver distraction: a naturalistic study of behaviour at intersections. Accid Anal Prev. 2013;58:271–278. doi:10.1016/j.aap.2012.12.027 23332726

[28] Owsley C, McGwin G Jr, Antin JF, Wood JM, Elgin J. The Alabama VIP older driver study rationale and design: examining the relationship between vision impairment and driving using naturalistic driving techniques. BMC Ophthalmol. 2018;18(1):32. doi:10.1186/s12886-018-0686-5 29415670 PMC5804048

[29] Swain TA, McGwin G Jr, Wood JM, Antin JF, Owsley C. Naturalistic driving techniques and association of visual risk factors with at-fault crashes and near crashes by older drivers with vision impairment. JAMA Ophthalmol. 2021;139(6):639–645. doi:10.1001/jamaophthalmol.2021.0862 33914022 PMC8085760

[30] Beck RW, Moke PS, Turpin AH, et al. A computerized method of visual acuity testing: adaptation of the early treatment of diabetic retinopathy study testing protocol. Am J Ophthalmol. 2003;135(2):194–205. doi:10.1016/s0002-9394(02)01825-1 12566024

[31] Pelli DG, Robson JG, Wilkins AJ. The design of a new letter chart for measuring contrast sensitivity. Clin Vis Sci 1988;2:187–199.

[32] Elliott DB, Bullimore MA, Bailey IL. Improving the reliability of the Pelli–Robson contrast sensitivity test. Clin Vis Sci. 1991;6:471–475.

[33] Leat SJ, Legge GE, Bullimore MA. What is low vision? A re-evaluation of definitions. Optom Vis Sci. 1999;76(4):198–211. doi:10.1097/00006324-199904000-00023 10333182

[34] Huisingh C, McGwin G Jr, Wood J, Owsley C. The driving visual field and a history of motor vehicle collision involvement in older drivers: a population-based examination. Invest Ophthalmol Vis Sci. 2014;56(1):132–138. doi:10.1167/iovs.14-15194 25395488 PMC4288142

[35] Kwon M, Huisingh C, Rhodes LA, McGwin G Jr, Wood JM, Owsley C. Association between glaucoma and at-fault motor vehicle collision involvement among older drivers: a population-based study. Ophthalmology. 2016;123(1):109–116. doi:10.1016/j.ophtha.2015.08.043 26459997 PMC4695303

[36] Nelson-Quigg JM, Cello K, Johnson CA. Predicting binocular visual field sensitivity from monocular visual field results. Invest Ophthalmol Vis Sci. 2000;41(8):2212–2221. 10892865

[37] Vargas-Martín F, García-Pérez MA. Visual fields at the wheel. Optom Vis Sci. 2005;82(8):675–681. doi:10.1097/01.opx.0000175624.34252.73 16127332

[38] Lacherez P, Au S, Wood JM. Visual motion perception predicts driving hazard perception ability. Acta Ophthalmol. 2014;92(1):88–93. doi:10.1111/j.1755-3768.2012.02575.x 23025481

[39] Swain TA, McGwin G Jr, Wood JM, Owsley C. Motion perception as a risk factor for motor vehicle collision involvement in drivers ≥ 70 years. Accid Anal Prev. 2021;151:105956. doi:10.1016/j.aap.2020.105956 33444870 PMC7878317

[40] Edwards JD, Ross LA, Wadley VG, et al. The useful field of view test: normative data for older adults. Arch Clin Neuropsychol. 2006;21(4):275–286. doi:10.1016/j.acn.2006.03.001 16704918

[41] Friedman C, McGwin G Jr, Ball KK, Owsley C. Association between higher order visual processing abilities and a history of motor vehicle collision involvement by drivers ages 70 and over. Invest Ophthalmol Vis Sci. 2013;54(1):778–782. doi:10.1167/iovs.12-11249 23307969 PMC3562119

[42] Reitan RM. The relation of the trail making test to organic brain damage. J Consult Psychol. 1955;19(5):393–394. doi:10.1037/h0044509 13263471

[43] Goode KT, Ball KK, Sloane M, et al. Useful field of view and other neurocognitive indicators of crash risk in older adults. J Clin Psychol Med Settings. 1998;5:425–440.

[44] Colarusso RP, Hammill DD. Motor-Free Visual Perception Test. Los Angeles, CA: Western Psychological Services; 1972.

[45] Ball KK, Roenker DL, Wadley VG, et al. Can high-risk older drivers be identified through performance-based measures in a Department of Motor Vehicles setting? J Am Geriatr Soc. 2006;54(1):77–84. doi:10.1111/j.1532-5415.2005.00568.x 16420201

[46] Folstein MF, Folstein SE, McHugh PR. “Mini-mental state”. A practical method for grading the cognitive state of patients for the clinician. J Psychiatr Res. 1975;12(3):189–198. doi:10.1016/0022-3956(75)90026-6 1202204

[47] Radloff LS. The CES-D scale: a self-report depression scale for research in the general population. App Psychol Meas. 1977;1:385–401. doi:10.1177/014662167700100306

[48] Owsley C, Stalvey B, Wells J, Sloane ME. Older drivers and cataract: driving habits and crash risk. J Gerontol A Biol Sci Med Sci. 1999;54(4):M203–M211. doi:10.1093/gerona/54.4.m203 10219012

[49] Dingus TA, Guo F, Lee S, et al. Driver crash risk factors and prevalence evaluation using naturalistic driving data. Proc Natl Acad Sci U S A. 2016;113(10):2636–2641. doi:10.1073/pnas.1513271113 26903657 PMC4790996

[50] Virginia Tech Transportation Institute. *Researcher Dictionary for Safety Critical Event Video Reduction Data.* 2015.

[51] McKnight AJ, Simone GA, Weidman JR. Elderly Driver Retraining. Springfield, VA: National Highway Traffic Safety Administration, US Department of Transportation; 1982.

[52] Owsley C, McGwin G Jr, Phillips JM, McNeal SF, Stalvey BT. Impact of an educational program on the safety of high-risk, visually impaired, older drivers. Am J Prev Med. 2004;26(3):222–229. doi:10.1016/j.amepre.2003.12.005 15026102

[53] Marottoli R, Van Ness P, Araujo K, et al. A randomized trial of an education program to enhance older driver performance. J Gerontol A Biol Sci Med Sci. 2007;62a:1113–1119. doi:10.1093/gerona/62.10.1113 17921424

[54] Bédard M, Porter MM, Marshall S, et al. The combination of two training approaches to improve older adults’ driving safety. Traffic Inj Prev. 2008;9(1):70–76. doi:10.1080/15389580701670705 18338298

[55] Coxon K, Hunter K, Chevalier A, et al. Behind the wheel: process evaluation of a safe-transport program for older drivers delivered in a randomized controlled trial. J Appl Gerontol. 2020;39(9):954–965. doi:10.1177/0733464818811015 30466338

[56] Stalvey BT, Owsley C. The development and efficacy of a theory-based educational curriculum to promote self-regulation among high-risk older drivers. Health Promot Pract. 2003;4(2):109–119. doi:10.1177/1524839902250757 14610980

[57] Owsley C, McGwin G Jr. Vision and driving. Vision Res. 2010;50(23):2348–2361. doi:10.1016/j.visres.2010.05.021 20580907 PMC2975746

[58] Owsley C, Ball K, McGwin G Jr, et al. Visual processing impairment and risk of motor vehicle crash among older adults. JAMA. 1998;279(14):1083–1088. doi:10.1001/jama.279.14.1083 9546567

[59] Rubin GS, Ng ES, Bandeen-Roche K, Keyl PM, Freeman EE, West SK. A prospective, population-based study of the role of visual impairment in motor vehicle crashes among older drivers: the SEE study. Invest Ophthalmol Vis Sci. 2007;48(4):1483–1491. doi:10.1167/iovs.06-0474 17389475

[60] Owsley C, Stalvey BT, Wells J, Sloane ME, McGwin G Jr. Visual risk factors for crash involvement in older drivers with cataract. Arch Ophthalmol. 2001;119(6):881–887. doi:10.1001/archopht.119.6.881 11405840

